# 47745 Low-risk Adenoma Surveillance Decision-making: Perspectives from Patients and Providers

**DOI:** 10.1017/cts.2021.548

**Published:** 2021-03-30

**Authors:** Jennifer K. Maratt, Marianne Matthias, Tayler Gowan, Thomas Imperiale

**Affiliations:** 1 Indiana University School of Medicine, Richard L. Roudebush VA Medical Center, Regenstrief Institute, Inc.; 2 Richard L. Roudebush VA Medical Center, Indiana University, Regenstrief Institute, Inc.; 3 Regenstrief Institute, Inc.

## Abstract

ABSTRACT IMPACT: I hope that our work will improve surveillance endoscopy experiences, by engaging both patients and providers. OBJECTIVES/GOALS: A large proportion of colonoscopies are performed for post-polypectomy surveillance. Data show that there is overuse of surveillance for low-risk adenomas (LRAs), which can be attributed to patient and provider factors. The objective is to understand patient and provider perspectives for decision-making for LRA surveillance colonoscopy. METHODS/STUDY POPULATION: Semi-structured, one-on-one virtual interviews of patients and providers are currently being conducted at Richard L. Roudebush VA Medical Center and Eskenazi Health. Using a criterion sampling approach, we identified patients 50-75 years of age who had a screening colonoscopy with finding of LRAs, and providers in primary care and gastroenterology, at each site. We plan to recruit at least 8 patients and 8 providers from each site until thematic saturation. Domains that will be covered include: perceived involvement with surveillance decision-making; experiences with, and preferences for, communication about test results; and barriers and facilitators to undergoing colonoscopy. A 3-phase approach, comprising immersion, reduction, and interpretation, is being used to collect and analyze data. RESULTS/ANTICIPATED RESULTS: This study is currently in the recruitment phase and results will be forthcoming. DISCUSSION/SIGNIFICANCE OF FINDINGS: Understanding decision-making for LRA surveillance colonoscopy will inform future interventions to improve endoscopic resource use and efficiency while improving patient and provider experiences with endoscopic care coordination.

